# Practical approach to diastolic dysfunction in light of the new guidelines and clinical applications in the operating room and in the intensive care

**DOI:** 10.1186/s13613-018-0447-x

**Published:** 2018-10-29

**Authors:** F. Sanfilippo, S. Scolletta, A. Morelli, A. Vieillard-Baron

**Affiliations:** 1 Department of Anesthesia and Intensive Care, IRCCS-ISMETT (Istituto Mediterraneo per i Trapianti e Terapie ad alta specializzazione), Palermo, Italy; 2 Unit of Intensive Care Medicine, Department of Medical Biotechnologies, University of Siena, Siena, Italy; 3 Department of Anaesthesiology and Intensive Care, University of Rome, “La Sapienza”, Rome, Italy; 4 Hospital Ambroise Paré, Assistance Publique-Hôpitaux de Paris, Boulogne, France

**Keywords:** Diastolic function, Systolic function, Weaning failure, Sepsis, Critical care

## Abstract

There is growing evidence both in the perioperative period and in the field of intensive care (ICU) on the association between left ventricular diastolic dysfunction (LVDD) and worse outcomes in patients. The recent American Society of Echocardiography and European Association of Cardiovascular Imaging joint recommendations have tried to simplify the diagnosis and the grading of LVDD. However, both an often unknown pre-morbid LV diastolic function and the presence of several confounders—i.e., use of vasopressors, positive pressure ventilation, volume loading—make the proposed parameters difficult to interpret, especially in the ICU. Among the proposed parameters for diagnosis and grading of LVDD, the two tissue Doppler imaging-derived variables *e*′ and *E*/*e*′ seem most reliable. However, these are not devoid of limitations. In the present review, we aim at rationalizing the applicability of the recent recommendations to the perioperative and ICU areas, discussing the clinical meaning and echocardiographic findings of different grades of LVDD, describing the impact of LVDD on patients’ outcomes and providing some hints on the management of patients with LVDD.

## Background

The study of left ventricular (LV) diastolic function and the impact of decreased LV compliance and impaired relaxation has received growing interest. This is certainly due to not only the high incidence of LV diastolic dysfunction (LVDD) in the general population and its sensible impact on patients outcomes [1], but also the growing use of echocardiography, which remains the sole clinical tool allowing the estimation of LV diastolic function. A cross-sectional survey of over 2000 randomly selected Minnesota residents aged 45 years or older found an incidence of LVDD almost five times higher than LV systolic dysfunction (28 vs. 6%, respectively), which was a strong predictor of mortality (hazard ratio ranging from 8.3 for mild LVDD to 10.2 for at-least-moderate LVDD) [1]. Up to 50% of patients presenting to the hospital with pulmonary edema and hypertension have unchanged LV systolic function and normal mitral valve apparatus, when compared during and after the acute episode [2]. Similarly, the incidence of isolated LVDD may be higher than 50% in patients hospitalized for heart failure (HF) [3].

The importance of LVDD is strongly emerging in the perioperative setting [4] and in critically ill patients [5–8], and the present review highlights the knowledge in the field. Moreover, since pharmacological strategies for improving LV diastolic function are limited and are more likely to produce results only in the long term [9], we provide a focused summary of the literature followed by key approaches that may help clinicians in the optimization and management of the patient with LVDD.

## Main text

### Recent guidelines and their applicability in the perioperative and intensive care settings

The most recent American Society of Echocardiography and European Association of Cardiovascular Imaging (ASE/EACVI) joint recommendations for the diagnosis and assessment of LVDD [10] made substantial changes compared with the previous recommendations [11]. One of the main aims of the new guidelines was to simplify the approach of clinicians to grading of LVDD, which in the previous version was deemed too complex because many parameters were included. Such recently revised guidelines have changed the methodology for determining LVDD, recommending an assessment mainly based on the following four variables: tricuspid regurgitation (TR) jet velocity, left atrial (LA) volume, *e*′ wave and *E*/*e*′ ratio. The *e*′ and *E*/*e*′ ratio are two parameters derived by tissue Doppler imaging (TDI) analysis, measuring longitudinal fiber lengthening during early diastole at level of mitral valve annulus using a modified pulsed wave Doppler setting (high amplitude, low velocity). The *e*′ maximal velocity reflects LV relaxation rate, while *E*/*e*′ correlates with the LV filling pressures and a ratio above 13–15 is associated with pulmonary arterial occlusion pressures > 18 mmHg [10]. The cutoffs for the four variables recommended by the guidelines are summarized in Table 1.

**Table 1:** Four main parameters used to define left ventricular diastolic dysfunction and their cutoffs

Parameter	Abnormal value	Mode of measurement	Limitation/confounders
TR jet velocity	> 2.8 m/s	Parasternal and apical 4-ch view with CFD to get highest velocity aligned with CWD. Adjust gain and contrast to display complete spectral envelope (no signal spikes or feathering) Analysis: peak modal velocity during systole at leading edge of spectral waveform	Indirect estimate of LA pressure; adequate recording of full envelope not always possible; in some cases accuracy of calculation is dependent on reliable estimation of right atrial systolic pressure
LA volume	> 34 mL/m^2^	Apical 4-ch and 2-ch: acquire freeze frames (1-2 frames before MV opening). LA volume measured in dedicated views (length and transverse diameters maximized) Analysis: method of disks or area-length method; correct for body surface area. Do not include LA appendage or pulmonary veins in tracings	LA dilatation is seen in bradycardia, high-output states, heart transplants, atrial flutter/fibrillation, significant MV disease, despite normal LV diastolic function; LA dilatation occurs in well-trained athletes; suboptimal image quality (i.e., foreshortening) precludes accurate tracings; it can be difficult to quantify in patients with aortic aneurysms or in patients with large inter-atrial septal aneurysms
*e*′	Septal < 7 cm/s Lateral < 10 cm/s	Apical 4-ch view: PWD sample volume (usually 5–10 mm axial size) at lateral or septal basal regions. Use ultrasound system presets for wall filter and lowest signal gain. Optimal spectral waveforms should be sharp (no signal spikes, feathering or ghosting) Analysis: peak modal velocity in early diastole at the leading edge of spectral waveform	Limited accuracy in patients with CAD and RWMAs, significant MAC, surgical rings or prosthetic MV, pericardial disease; need to sample at least two sites; different cutoffs depending on sampling site; age dependent (decreases with aging)
*E*/*e*′ ratio	Average > 14 Septal > 15 Lateral > 13	E wave: apical 4-ch with CFD imaging for optimal alignment of PWD with blood flow. PWD sample volume (1–3 mm axial size) between mitral leaflet tips. Use low wall filter setting (100–200 MHz) and low signal gain. Optimal spectral waveforms should not display spikes or feathering Analysis: peak modal velocity in early diastole at the leading edge of spectral waveform *e*′: see above Analysis: E velocity divided by *e*′ velocity	Not accurate in normal subjects, patients with MAC, pericardial disease; “gray zone” of values in which LV filling pressures are indeterminate; accuracy reduced in CAD and RWMAs; different cutoff values depending on the site used for measurement

4-ch, four-chamber; 2-ch, two-chamber; CAD, coronary artery disease; CFD, color flow Doppler; CWD, continuous wave Doppler; LA, left atrium; LV, left ventricle; MAC, mitral annulus calcifications; MV, mitral valve; PWD, pulsed wave Doppler; RWMAs regional wall motion abnormalities; TR, tricuspid regurgitation

In patients with normal LV systolic function, abnormalities of more than half of measurable parameters (i.e., a patient may have no TR) define the presence of LVDD. On the other hand, the new guidelines support that patients with structural abnormalities, known ischemic heart disease or abnormal LV systolic function will have impaired myocardial relaxation, and thus, echocardiography examination may focus on the assessment of LV filling pressures and diastolic dysfunction grade.

Once diagnosis of LVDD is made, the following step is to proceed with grading of the dysfunction itself. The four parameters indicated in Table 1 and the *E*/*A* ratio are used to grade LVDD and can be found in the recently published recommendations [10], but LVDD grading is not the aim of the present review, which is not intended for cardiologists or experts in echocardiography. Of note, an interesting retrospective cohort study by Almeida and Colleagues conducted on 1000 individuals aged ≥ 45 years and with normal systolic function found poor concordance between the new and the previous versions of the guidelines (published in 2009) for the evaluation of LV diastolic function [10, 11]. In this study, the new guidelines resulted in a significantly lower incidence of LVDD (1.4 vs. 38.1% of 2009 recommendations) [12].

Unfortunately, assessing LVDD is not always easy and the guidelines’ authors themselves state “…*the guidelines are not necessarily applicable to children or in the perioperative setting.”* The fact that applicability of these guidelines to the perioperative setting represents a challenge is not surprising. Indeed, patients undergoing major surgery are mechanically ventilated and exposed to drugs with vasoactive effects, and they easily fluctuate from hyper- to hypovolemia due to perioperative fasting, fluid shift, hemorrhage, etc. Moreover, the use of transthoracic echocardiography (TTE) is limited in the operating room, and the applicability of *e*′ and *E*/*e*′ ratio with transesophageal echocardiography (TEE) should consider the importance of obtaining a good alignment of TDI signal.

On the other hand, it is somewhat surprising that the authors did not mention the limitations of such new guidelines in the critically ill patients. Same limitations as the ones described in the perioperative setting may be also present. Moreover, in this population of patients, the TR jet velocity may worsen under the negative influence of mechanical ventilation on right ventricular (RV) function and there are a large number of factors that may increase pulmonary pressure at pre-capillary level, making the reliability of TR jet velocity at least questionable. Among these factors, pulmonary vascular resistances may increase with elevated airway pressures, although it should be kept in mind that the transmission of pleural pressure itself is reduced when lung compliance is low (i.e., acute respiratory distress syndrome). Additionally, the effects of mechanical ventilation on LV diastolic function are not negligible, and one study in cardiac surgery patients showed that increasing levels of positive end-expiratory pressure (PEEP) reduced significantly both septal and lateral *e*′ values, possibly representing an impaired LV relaxation due to worse RV function (and possibly RV dilatation) [13]. In other words, the observed LVDD may not be related to the disease itself, but driven by the conditions of mechanical ventilation. One could say that LVDD is associated with a worst prognosis when reflecting a specific injury of the myocardium, while probably this is not the case when LVDD is mainly induced by ventilation settings and clinical management.

With respect to the second parameter listed in Table 1, the LA volume is influenced by loading conditions and critically ill patients are certainly exposed to sudden changes of circulating volume for either absolute or relative hypovolemia (i.e., trauma and/or sepsis). More importantly, patients with sepsis or septic shock are characterized not only by vasoplegia with reduction in LV afterload, but they also show myocardial depression (septic cardiomyopathy) [14]. Furthermore, as clarified in the recent guidelines, LA enlargement is observed when LVDD is chronic and cannot probably be used in more acute situations, as frequently observed in the intensive care unit (ICU). How acutely the LA can dilate during the early stages of critical illness is a matter of future research. A recent study evaluated early changes in LA volume after acute myocardial infarction. At 4-month follow-up the authors found that 35% of patients had LA remodeling, defined as LA volume index ≥ 10 ml/m^2^. However, at 1-month follow-up there was a mean change of 6 ml/m^2^, with no significant differences between patients with or without LA remodeling [15]. Therefore, it seems that changes in LA volume can happen in a relatively short-term but not so acutely as it matters in the case of critically ill patients. Interestingly, another study demonstrated that magnitude and pattern of LA appendage emptying/filling velocities are dependent on loading conditions and that velocities are influenced mainly by changes in LV rather than in LA appendage function [16]. In light of the above, estimation of LA volume as for the prediction of acute changes of LV diastolic function in critically ill patients seems a physiologically imprecise parameter. Moreover, it is important to note that the LA volume is not precisely quantifiable with TEE, which adds further limitations for patients necessitating an echocardiographic assessment but having poor acoustic windows for a transthoracic examination, for whatever reason (i.e., due to mechanical ventilation). Another significant issue in the ICU regards the inability of echocardiographic evaluation to diagnose whether the LVDD is a new acute finding, mainly related to the critical illnes (e.g. sepsis for instance) and then possibly reversible, or if LVDD pre-existed to the ICU admission, considering the amount of admission of older and older patients carrying a significant burden of comorbidities. The only way to differentiate between both is to repeat echocardiographic evaluation longitudinally until the discharge and maybe after full recovery, but this approach would be very time- and resource-consuming. However, observing a LA dilatation could help physicians determine whether diastolic dysfunction existed prior to admission in the ICU.

For the above reasons it becomes challenging the assessment of sepsis-related changes in LV diastolic function according to fluctuations in LA volume and/or TR jet velocities, while the two TDI parameters (*e*′ and *E*/*e*′, see Table 1) probably remain the only reliable approach, due to their relative independency from the loading state [17]. Importantly, a recent meta-analysis by Sanfilippo et al. [5] showed that such parameters are associated with survival in septic patients.

### Clinical and echocardiographic findings of different grades of LVDD

**Fig. 1:**
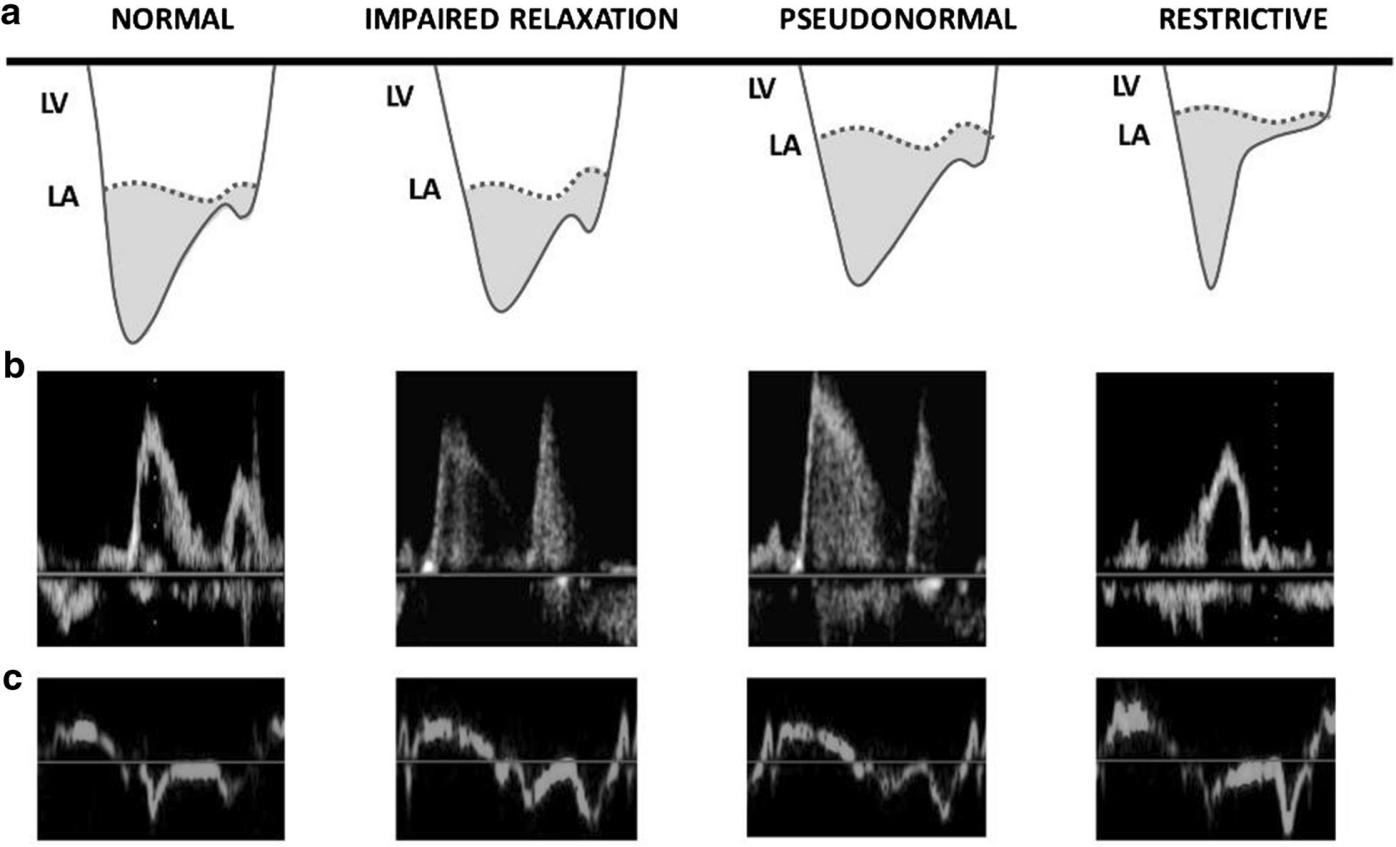
Progression from normal diastolic function to worsening degrees of left ventricular diastolic dysfunction (LVDD). The top row **a** illustrates the respective changes in left atrial (LA) and left ventricular (LV) pressures with the progression of LVDD. The middle and bottom rows show examples of the patterns of transmitral blood flow (**b**) and of tissue Doppler imaging of the mitral annulus (**c**). These patterns are shown for each stage of LVDD, with corresponding changes of the *E* and *e*′ (early), and *A* and *a*′ (atrial) waves. From left to right, 2a: normal diastolic function (*E* > *A*; *e*′ > *a*′); 2b: LVDD grade I (*E* < *A*; *e*′ < *a*′); 2c: LVDD grade II (*E* > *A*; *e*′ < *a*′); 2d: LVDD grade III (*E* ≫ *A*; *e*′ ≪ *a*′)

**Fig. 2:**
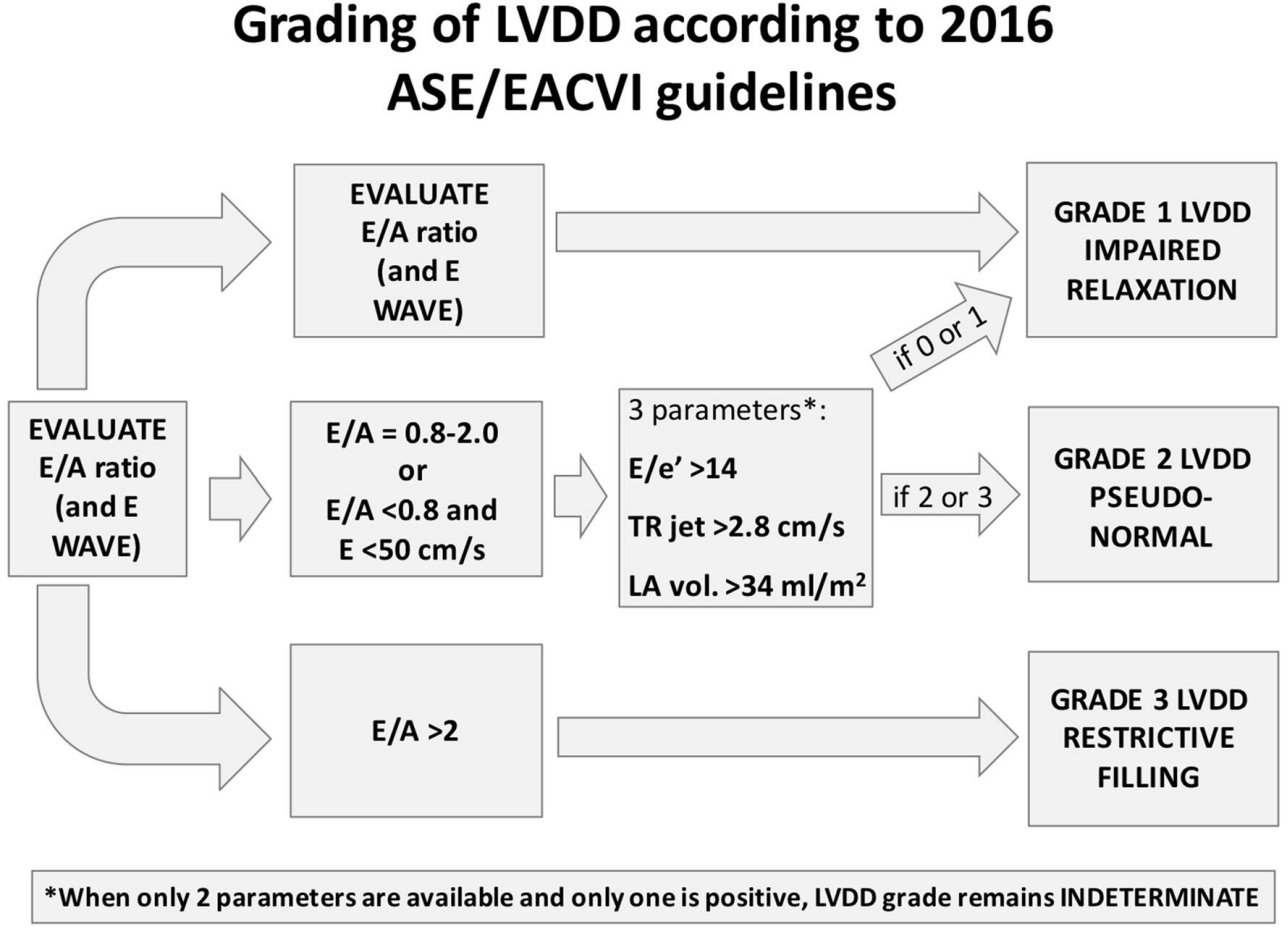
Algorithm for grading of left ventricular diastolic dysfunction (LVDD) in outpatients according to the 2016 American Society of Echocardiography and European Association of Cardiovascular Imaging (ASE/EACVI) guidelines

Hereby, we provide a simplified interpretation to the progression from normal LV diastolic function to different degrees of LVDD. In this context, it should be kept in mind that LV diastolic properties and LV filling pressure are closely related. In particular, Fig. 1 shows the relationship between left-sided pressures (LA and LV, Fig. 1a) and the corresponding echocardiographic findings for each stage in terms of transmitral blood flow (Fig. 1b) and of TDI mitral annular displacement (Fig. 1c). In the outpatient setting, once diagnosis of LVDD is made (i.e., patients with reduced LV systolic function and/or structural cardiomyopathy and/or fulfilling 3–4 parameters shown in Table 1), the grading of dysfunction is assessed according to the *E/A* ratio (and eventually *E* wave velocity). Figure 2 shows an algorithm for grading of LVDD in the outpatients according to the ASE/EACVI 2016 guidelines.

From physiological perspectives, in patients with normal LV diastolic function, the LV fills smoothly in the presence of low LA pressures and thus with a relatively small LA-to-LV gradient (in the order of few mmHg). The corresponding echocardiographic appearance in the transmitral blood flow is a dominant early (*E*) wave over the atrial (*A* or late) wave, and the corresponding TDI *e*′ and *a*′ waves demonstrate a similar ratio (first column from the left in Fig. 1).

With regard to the interpretation of transmitral blood flow, at initial stages of LVDD the LV becomes stiffer with impaired LV relaxation and the LA-to-LV gradient becomes smaller. Therefore, the early filling wave gradually decreases and the atrial wave becomes dominant (*E* < *A* wave): This is the classical features of LVDD grade I (second column from the left in Fig. 1).

The subsequent progression of LVDD with further relaxation impairment causes physiological mechanisms of adaptation (i.e., fluid retention and changed loading conditions) with a consequent increase in LA pressure in order to restore a “pseudo-normal” LA-to-LV gradient (LVDD grade II). Thus, during this stage of LVDD there is a “pseudo-normalization” of transmitral flow pattern (*E* > *A* wave) due to the reactive increase in LA pressures in response to worsening LVDD (third column from the left in Fig. 1).

Finally, when the LV chamber becomes poorly compliant and increasingly stiff (LVDD grade III), only a certain amount of blood can flow from the LA to the LV at each diastole. Importantly, such reduced amount of blood flowing into the LV during the early phase of diastole (*E* wave) quickly boosts the LV end-diastolic pressure at very high level so that the subsequent atrial contraction is unable to generate a decent filling (usually in the order of very few ml of blood). Consequently, the *E* wave is very dominant and the *E/A* ratio is usually > 2 (last column from the left in Fig. 1). The LVDD grade III has been further divided into reversible and irreversible, but performing such distinction is challenging, it requires patient’s collaboration (i.e., performing Valsalva manoeuver), and more importantly it is probably more useful in the cardiology outpatient setting rather than in the operating room or in the ICU patients.

On the other hand, as shown in Fig. 1c, changes in TDI waves *e*′ and *a*′ are more unidirectional with the development of LVDD. The *a*′ is an excellent marker of global atrial contraction and has similar values at septal and lateral levels [18]. It correlates very well with LA ejection fraction, LA ejection force and LA kinetic energy [19], being independent from the flow of blood filling the LV. For this reason the *a*′ does not become smaller, but rather increases with progression of LV diastolic dysfunction and with more vigorous LA contraction in adaptation to the increased pressures. Only with advanced LA dilatation reaching a threshold of fiber length, LA shortening and contractility begin to decline, similar to what happens for the LV (Frank–Starling curve) [20–23]. On the other hand, the decline in *A* wave is likely to happen earlier in the progression of LVDD, because it is related to the reduction in transmitral blood flow in the presence of very high LV filling pressure.

Therefore, there is a progressive decrease in *e*′ and a consequent increase in *a*′ so that the *e*′/*a*′ ratio moves gradually from > 1 to < 1 values; however, while *e*′/*a*′ > 1 usually denotes a normal LV diastolic function and *e*′/*a*′ ≪ 1 is of restrictive pattern, it is more difficult to use the *e*′/*a*′ ratio for the distinction between LVDD grades I and II.

In general, this paragraph provides a summary that may help readers in understanding the relationships between the LA-to-LV gradient and the changes in transmitral blood flow and mitral annular TDI displacement. It is mandatory to keep in mind that the interpretation of such parameters should take into account factors like patient’s history (i.e., chronic atrial fibrillation—AF—may cause LA enlargement) and physiological factors (i.e., age influences cutoff for *E* wave). Moreover, the assessment becomes even more challenging in the ICU where the echocardiographic parameters can be affected by several confounders. For instance, the heart rate (especially tachycardia) and the use of vasopressors and/or inotropes influence the LV diastolic properties; the *E/A* ratio may vary according to non-hemodynamic factors such as mechanical ventilation; and it is also of limited value in patients with significant mitral and/or aortic valve disease, or before fluid resuscitation has been carried out in critically ill patients.

### Impact of LVDD in the perioperative setting

Nowadays, surgery is performed without a true “age cutoff” with older and older patients undergoing surgical procedures. Such patients have a large burden of comorbidities, including LVDD. However, the vast majority of literature of LVDD in the perioperative setting includes patients undergoing cardiac surgery or vascular surgery, since these patients generally present a larger spectrum of comorbidities, especially from cardiovascular perspectives. Moreover, such patients are frequently monitored perioperatively with TEE and thus real-time estimation and monitoring of LVDD could be feasible, although it should be kept in mind that TDI measures have not yet been fully validated with TEE.

In patients undergoing cardiac surgery, LVDD correlates with difficult weaning from cardiopulmonary bypass and higher inotropic needs [24, 25]. Moreover, there is some evidence of correlation between advanced LVDD and postoperative AF after cardiac surgery [26, 27].

In patients undergoing major vascular surgery, preoperative isolated LVDD is more frequent than isolated LV systolic dysfunction (43 vs. 8%, respectively) and, importantly, LVDD is an independent predictor of postoperative HF and prolonged hospital stay, and it is associated with postoperative adverse cardiovascular events and long-term cardiovascular mortality [28, 29].

While the impact of preoperative LVDD in these high-risk surgical specialties is not unexpected, more uncertainty reigns on the importance of LVDD in patient’s outcome in other surgical specialties. In this regard, one of the issues is the ethical concerns and potential value in performing intraoperative TEE in patients with isolated LVDD undergoing non-high-risk surgery. Cabrera-Schulmeyer and Arriaza conducted an interesting study in patients with cardiac comorbidities and undergoing non-cardiac/non-vascular (abdominal, urological and orthopedic) surgery. The authors stratified patients according to preoperative normal (< 8), borderline (8–15) and high (> 15) *E*/*e*′ ratio. Patients with borderline and high *E*/*e*′ had a higher incidence of perioperative complications (higher incidence of pulmonary edema at 24 h and 48 h, arrhythmias) and longer ICU stay and hospital stay than patients with normal *E*/*e*′. Moreover, patients with high *E*/*e*′ had significantly higher mortality as compared to normal ratio (8 vs. 0%, respectively) [4].

While preoperative LVDD correlates with outcome, a postoperative evaluation of patient’s diastolic function should consider ruling out first the influence of stressors that may worsen diastolic function (i.e., pain-related tachycardia reduces diastolic time, hypo- and hypervolemia may influence LV filling pressures, etc.).

### Impact of LVDD in the intensive care

With respect to the role of LVDD in (non-cardiac surgery) critically ill patients, the greater amount of research is related to the role of LVDD in the outcome of sepsis and in the weaning from mechanical ventilation.

### LVDD and sepsis

Septic shock is characterized by intense vasoplegia requiring vasoactive therapy to restore blood pressure [30]; however, it has become more evident over the past years that septic patients are affected by pronounced myocardial dysfunction, which is possibly the result of increased circulating cytokine and catecholamine levels [14, 31]. We emphasize that, in case of overt septic shock and in the absence of signs of other causes of shock, fluid resuscitation should not be delayed in order to get information on LV diastolic function from an advanced critical care echocardiography examination or by requesting cardiology consultation. It is also likely that in patients with pronounced hypovolemia (and vasoplegia), the parameters used for the evaluation of LV diastolic function will undergo dramatic changes according to fluid resuscitation and/or the start of vasopressor infusion. It is pivotal understanding that assessment of LV diastolic function cannot be dissociated from evaluation of LV filling pressure, which in certain group of patients undergoes sudden clinical variations.

The so-defined septic cardiomyopathy may involve either the LV, the RV, or both, affecting systolic and/or diastolic function. With all the limitations coming from the use of LV ejection fraction (LVEF) for the evaluation of systolic function, a meta-analysis found no association between LV or RV systolic dysfunction and mortality in patients with severe sepsis or septic shock [32]. On the other hand, a subsequent meta-analysis demonstrated a strong association between LVDD and mortality in the same population of critically ill patients and confirmed also the absence of association between LV systolic dysfunction and mortality [6, 7]. Moreover, the same group of authors recently showed that worse TDI parameters (lower *e*′ and higher *E*/*e*′ ratio) are associated with mortality in septic patients [5]. Interestingly, another meta-analysis investigated the value of speckle tracking echocardiography in the prognostic evaluation of septic cardiomyopathy, showing that worse values of LV strain are associated with negative outcome in septic patients [33]. More research is warranted for speckle tracking echocardiography to understand if it could represent a better marker of intrinsic LV function in critically ill patients.

The association between LVDD and outcome in patients with severe sepsis and septic shock may be explained looking at the pathophysiology of sepsis. In patients with LVDD, the LV filling benefits from maintenance of adequate preload, sinus rhythm and avoidance of tachycardia, while sepsis causes sequential disturbances at such levels since patients become relatively hypovolemic, tachycardic and frequently develop arrhythmias, with AF described in up to 23% of patients with septic shock [34, 35]. Septic patients are relatively hypovolemic due to vasoplegia and increased capillary permeability and higher venous capacitance. In fact, the recommended first-line therapy for the treatment of sepsis is to restore preload. In patients with LVDD and increased LV filling pressures there is probably a narrow window for optimizing fluid status. In these patients, even under condition of theoretical “fluid-responsiveness,” a little amount of fluid may precipitate pulmonary edema or cause a hemodynamic collapse. Therefore, without any delay in the initial fluid resuscitation of septic patients, the subsequent preload optimization may benefit by the knowledge of his/her LVDD conditions, too, which should be integrated with other variables. For instance, patients with acute respiratory distress syndrome and/or acute or pulmonale may not benefit from fluid due to both hydrostatic worsening of non-cardiogenic pulmonary edema and further RV dilatation. Patients with severe RV dysfunction may suffer from extra amount of fluids because the RV dilatation together with paradoxical septal motion hampers LV filling creating a “LVDD-like” condition by pushing the septal region and reducing LV compliance. However, using an experimental model of lung injury and high airway pressures, Katira et al. [36] reported very low augmentation of LV filling pressure as a consequence of RV failure, possibly as a consequence of decreased venous return and/or increased pulmonary vascular permeability.

Similarly, patients with at-least-moderate mitral regurgitation may worsen their pulmonary function if an extra amount of fluid is administered [37]. Not only preload but afterload too affects negatively the evaluation of LV diastolic function. In this regard, LV diastolic function worsens as a consequence of increased afterload due to hypertension [38] or related pharmacological [39] and non-pharmacological (increased intra-abdominal pressure [40]) factors. However, the main issues in this matter are represented by the difficulty of directly and reliably quantifying the LV afterload with echocardiography.

Another factor that further worsens LV filling is tachycardia, mainly disproportionally reducing the LV diastolic time. Although healthy individuals compensate for by accelerating the LV relaxation process (frequency-dependent acceleration of relaxation [41]), this process is impaired during sepsis [42]. The LV filling is further worsened by the development of AF and the consequent loss of efficacious atrial contraction. In this respect, although speculative, it is possible that the use of beta-blockade may produce more benefits in septic patients with LVDD for their ability to reduce heart rate and for their anti-arrhythmic properties [43, 44]. Another hypothesis which has to be evaluated in the future is the fact that septic patients with LVDD may have a worst tolerance to fluid expansion.

Concerning the incidence of LVDD in sepsis, it is worth noting that a recent study by Clancy et al. [45] found that the application of new guidelines for the evaluation of LV diastolic function identified a significantly higher incidence of LVDD as compared to the previous 2009 guidelines. This finding is interesting since a study in the general population found opposite results (much lower incidence of LVDD with new guidelines as compared to the 2009 version), as previously discussed [12].

### LVDD and weaning from mechanical ventilation

During mechanical ventilation, LV preload and afterload are decreased, and the transition from positive to negative pressure (spontaneous breathing) creates unfavorable LV loading conditions and may also trigger myocardial ischemia [46]. There is growing evidence that the largest amount of weaning failures are of cardiac origin.

A recent study investigated the value of a combined integrated thoracic sonographic evaluation (respiratory, cardiac and diaphragmatic) in predicting early post-extubation respiratory distress. The detection of lung interstitial water was the most relevant parameter detected during thoracic ultrasound, while among factors studied by echocardiography, the estimation of LV filling pressure was predictive of post-extubation distress. On the contrary, indexes of systolic function and diaphragmatic excursion had poor impact over the prediction of respiratory failure [47].

One study showed an association between weaning failure and both lower LVEF and higher *E*/*e*′ [48], but another one failed to show an association between LVEF and weaning failure [49]. The presence of LVDD seems more strongly associated with weaning failure as shown by several studies. Konomi et al. [50] found an independent association between LVDD and weaning failure (odds ratio 11.2), while Papanikolaou et al. [51] found lateral *E*/*e*′ as the only factor independently associated with weaning failure possibly reflecting the association between a higher degree of LVDD and weaning failure. Moschietto et al. [49] found higher *E*/*e*′ and lower *e*′ in the failing group and interestingly that *e*′ velocity increased in patients successfully weaned, while it remained unchanged in those failing.

The largest study on this topic recently showed that the vast majority of patients failing a spontaneous breathing trial (SBT) and weaning from mechanical ventilation develop weaning-induced pulmonary edema (WiPO) and that structural cardiopathy, chronic obstructive pulmonary disease and obesity are the main risk factors for WiPO [52]. In a subgroup of patients with cardiac output monitoring, the authors were able to show that WiPO is associated with *preload*-*independence* and that a subsequent SBT is more likely to succeed after diuretic therapy and a more negative fluid balance (achieving *preload*-*dependence*). This study found similar LVEF and fluid balance between patients with WiPO and non-WiPO, but the first group had significantly higher *E*/*e*′ ratio, possibly reflecting a worse diastolic function [8]. In support of this hypothesis, WiPO failure patients monitored with cardiac output showed an increase in both global end-diastolic volume and extra-vascular lung water as compared to non-WiPO failures where these remained unchanged. Such findings highlight the risk of the transition from positive to negative pressure ventilation, where an increase in LV preload cannot be accommodated in patients with high LV filling pressures.

## How to manage the patient with LVDD

**Fig. 3:**
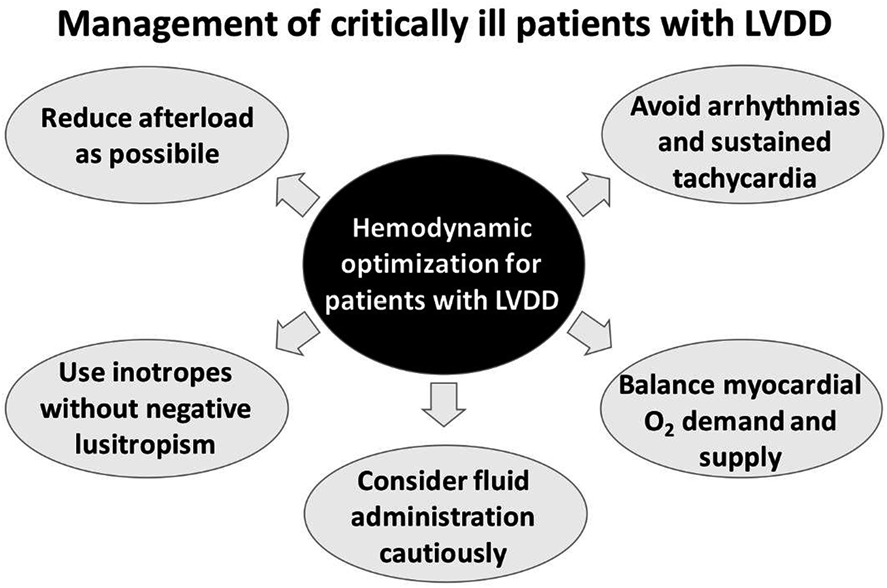
Suggestions for the management of critically ill patients with left ventricular diastolic dysfunction (LVDD)

The management of patients with LVDD can be challenging, and unfortunately, there is no magic bullet that rapidly improves LV diastolic function, pharmacologically or non-pharmacologically. A graphical summary of suggestions to manage the critically ill patients with LVDD is provided in Fig. 3. Moreover, no study exists to demonstrate that improving LVDD in the critically ill patients could beneficially impact the prognosis. Only few drugs have shown some improvements of LVDD. Among them beta-blockers are an example. Indeed, it is known their ability to ameliorate LVDD in HF with preserved LVEF [9], and beta-blockade also improves LV filling pressures and coronary flow reserve in patients with uncomplicated arterial hypertension [53]. It is worth noting the results of the first large randomized study on beta-blockers in patients with septic shock, with beneficial effects of esmolol infusion as shown by a significant improvement in cardiac performance, lower inotropic requirements and higher survival as compared to placebo. Both negative chronotropic and anti-arrhythmic effects of esmolol may have had positive influence on LV diastolic function, although the authors did not present echocardiographic data and this hypothesis remains speculative.The same group of authors recently showed an improved LV filling pattern and ventriculo-arterial coupling after esmolol infusion in septic patients [54]; moreover, immune, metabolic and coagulative effects of beta-blockers treatment may result advantageous in patients with sepsis [44]. Another therapeutically plausible option for patients with heart failure could be represented by the use of angiotensin-converting enzyme inhibitors, which may reduce LV remodeling and improve diastolic function [55–58]. However, the effects of this class of drugs are evident in the long run only and their efficacy is not demonstrated for LVDD in the acute setting. A potentially interesting drug that may ameliorate the hemodynamic profile of septic patients may be represented by dexmedetomidine (α-2 agonist), a sedative drug that has shown a possible reduction in catecholamines release associated with increased blood pressure response to exogenous vasopressors in experimental models of septic shock [59–62]. A clinical study has completed its enrollment (ClinicalTrials.gov Identifier: NCT02638545), but more research is warranted before drawing any conclusion. Another drug that has shown improvements of LV diastolic function is levosimendan [63], and its properties are unique if compared to catecholamines (which usually worsen LV diastolic function [64]) and phosphodiesterase inhibitors (LV diastolic function remaining grossly unchanged) [65]. However, levosimendan has specific pharmacokinetic and pharmacodynamic properties that make it not ideal when the effect is needed rapidly.

In general, although LVDD can be potentially reversed or reduced in its magnitude with appropriate treatment in the long run, this seems rather difficult in the acute critically ill patients; since the pharmacological approach to the optimization of diastolic function does not offer rapid solutions at present, clinicians should probably focus on the maintenance of the best loading conditions for the patient with established LVDD. From a clinical perspective, patients with LVDD grade I are usually more easy to manage, but the dominance of the atrial filling (*E* < *A*, see Fig. 1) makes them very sensible to the loss of atrial filling (i.e., AF) in case of baseline reduced LVEF. Therefore, particular attention should be devoted to the avoidance of AF [66].

In case of patients with LVDD grade II, clinicians should carefully evaluate the volume status. Indeed, such patients are prone to develop pulmonary edema under condition of hypervolemia; on the other hand, in case of hypovolemia the LA pressure decreases and patients lose their compensatory mechanism to maintain a “normal” LA-to-LV gradient, and this situation can be further aggravated by AF (loss of atrial contribution).

Patients with LVDD grade III are generally very frail patients and, for instance, severe abnormalities in LV filling pattern can explain the case of patients with HF with preserved LVEF, where patients are symptomatic despite no gross alteration in LVEF. In the outpatients, these individuals benefit from cardiology consultation and optimization, and they may also be considered for cardiac resynchronization therapy in case of prolonged QRS [67]. However, this option may not be easy in the acute phase of critical illness [68]. It is the authors’ opinion that, if an urgent intervention is needed, such patients should be possibly managed by anesthesiologists with experience in the cardiac setting and optimized using echocardiography across the perioperative period.

Finally, because diastolic function is affected early during myocardial ischemia (earlier than systolic function) [69], attention should be paid to the factors associated with myocardial hypoperfusion. Ensuring an appropriate oxygen delivery to the LV by maintaining adequate diastolic blood pressure (with careful approach especially in patients with reduced arterial elastance and with risk factors for—or known for—coronary artery disease), possibly reducing the heart rate and thus the myocardial oxygen demand, and balancing the right level of LV afterload should all be part of the clinician’s considerations when approaching the patient with LVDD.

## Conclusion

There is growing evidence on the contribution of diastolic function to patients’ outcome both in the perioperative setting and in the ICU. The assessment and grading of diastolic dysfunction remains challenging in these patients, and the guidelines used in the outpatient setting are not fully applicable. While pharmacological optimization remains difficult, especially with time constraints (urgency/emergency cases), a proactive management aiming at maintaining adequate loading conditions and an appropriate balance between myocardial oxygen demand and delivery could be the best strategies in managing patients with left ventricular diastolic dysfunction.

## Abbreviations

LV: left ventricle
LVDD: left ventricular diastolic dysfunction
HF: heart failure
ASE: American Society of Echocardiography
EACVI: European Association of Cardiovascular Imaging
TR: tricuspid regurgitation
LA: left atrium
TDI: tissue Doppler imaging
TTE: transthoracic echocardiography
TEE: transesophageal echocardiography
RV: right ventricle
PEEP: positive end-expiratory pressure
AF: atrial fibrillation
LVEF: left ventricular ejection fraction
SBT: spontaneous breathing trial
WiPO: weaning-induced pulmonary edema
