# Correction to: Practical approach to diastolic dysfunction in light of the new guidelines and clinical applications in the operating room and in the intensive care

**DOI:** 10.1186/s13613-018-0452-0

**Published:** 2018-11-06

**Authors:** F. Sanfilippo, S. Scolletta, A. Morelli, A. Vieillard-Baron

**Affiliations:** 1 Department of Anesthesia and Intensive Care, IRCCS-ISMETT (Istituto Mediterraneo per i Trapianti e Terapie ad alta specializzazione), Palermo, Italy; 2 Unit of Intensive Care Medicine, Department of Medical Biotechnologies, University of Siena, Siena, Italy; 3 Department of Anaesthesiology and Intensive Care, University of Rome, “La Sapienza”, Rome, Italy; 4 Hospital Ambroise Paré, Assistance Publique-Hôpitaux de Paris, Boulogne, France

## Correction to: Ann. Intensive Care (2018) 8:100 10.1186/s13613-018-0447-x

In the original article [1], the authors noticed a typographical error in Figure 2. The top left box should have included “E/A <0.8 and E <50 cm/s”. Please see below the corrected Fig. 2.

**Fig. 2:**
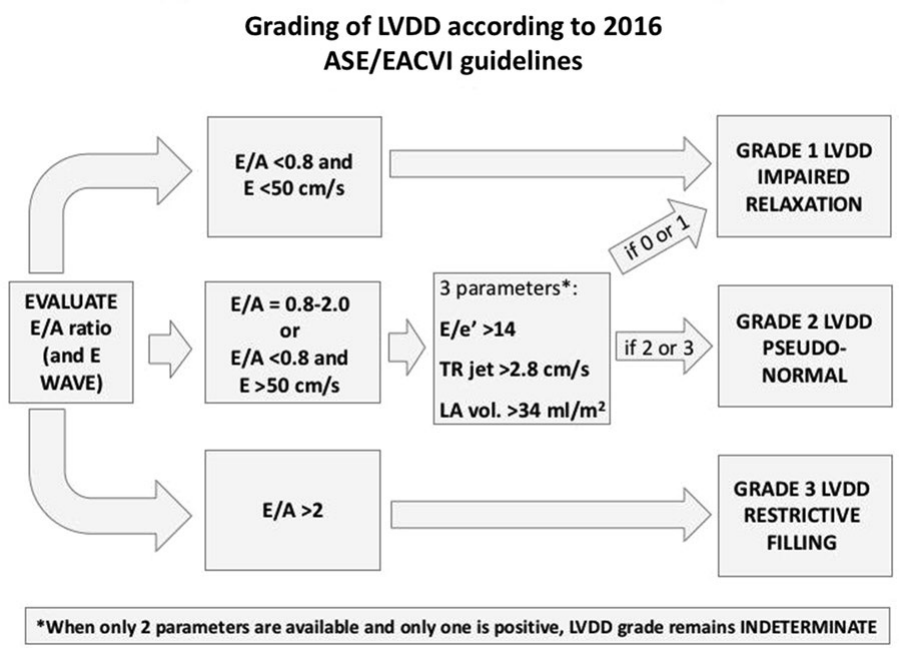
Algorithm for grading of left ventricular diastolic dysfunction (LVDD) in outpatients according to the 2016 American Society of Echocardiography and European Association of Cardiovascular Imaging (ASE/EACVI) guidelines
